# Health care utilization and health-related quality of life of injury patients: comparison of educational groups

**DOI:** 10.1186/s12913-021-06913-3

**Published:** 2021-09-19

**Authors:** Marjolein van der Vlegel, Inge Spronk, Joost Oude Groeniger, Hidde Toet, Martien J. M. Panneman, Suzanne Polinder, Juanita A. Haagsma

**Affiliations:** 1 Department of Public Health, Erasmus MC, University Medical Center Rotterdam, P.O. Box 2040, CA Rotterdam, 3000 The Netherlands; 2 Association of Dutch Burn Centres, Maasstad Hospital, Rotterdam, the Netherlands; 3 Consumer Safety Institute, Amsterdam, The Netherlands

**Keywords:** Health care utilization, Health-related quality of life, Educational differences, SES

## Abstract

**Background:**

Differences in health care utilization by educational level can contribute to inequalities in health. Understanding health care utilization and health-related quality of life (HRQoL) of educational groups may provide important insights into the presence of these inequalities. Therefore, we assessed characteristics, health care utilization and HRQoL of injury patients by educational level.

**Method:**

Data for this registry based cohort study were extracted from the Dutch Injury Surveillance System. At 6-month follow-up, a stratified sample of patients (≥25 years) with an unintentional injury reported their health care utilization since discharge and completed the EQ-5-Dimension, 5-Level (EQ-5D-5L) and visual analogue scale (EQ VAS). Logistic regression analyses, adjusting for patient and injury characteristics, were performed to investigate the association between educational level and health care utilization. Descriptive statistics were used to analyse HRQoL scores by educational level, for hospitalized and non-hospitalized patients.

**Results:**

This study included 2606 patients; 47.9% had a low, 24.4% a middle level, and 27.7% a high level of education. Patients with low education were more often female, were older, had more comorbidities, and lived more often alone compared to patients with high education (*p* < 0.001). Patients with high education were more likely to visit a general practitioner (OR: 1.38; CI: 1.11–1.72) but less likely to be hospitalized (OR: 0.79; CI: 0.63–1.00) and to have nursing care at home (OR: 0.66; CI: 0.49–0.90) compared to their low educated counterparts. For both hospitalized an non-hospitalized persons, those with low educational level reported lower HRQoL and more problems on all dimensions than those with a higher educational level.

**Conclusion:**

Post-discharge, level of education was associated with visiting the general practitioner and nursing care at home, but not significantly with use of other health care services in the 6 months post-injury. Additionally, patients with a low educational level had a poorer HRQoL. However, other factors including age and sex may also explain a part of these differences between educational groups. It is important that patients are aware of potential consequences of their trauma and when and why they should consult a specific health care service after ED or hospital discharge.

**Supplementary Information:**

The online version contains supplementary material available at 10.1186/s12913-021-06913-3.

## Background

Socioeconomic inequalities in health are an important challenge for public health [1, 2]. Economic, educational and occupational resources are viewed as components of socio-economic status (SES) and have found to be associated with health outcomes including morbidity and mortality [2]. Health inequalities can be defined as systematic, avoidable and unfair differences in health outcomes and exists on a gradient in social classes such as levels of educational attainment [3, 4]. Access to and quality of health care and education, employment and income are examples of social determinants of health. These social determinants of health shape health status and well-being [5]. Health related quality of life (HRQoL) is a health outcome which quantifies overall health and previous studies showed socio-economic inequalities in HRQoL in general populations [6]. It is important to examine socioeconomic inequalities in the utilization of health care, in order to shed a light on the role of health care in explaining health inequalities. Several studies, performed in countries with varying health care systems, investigated the effect of SES on health care utilization in general and multi-morbidity populations [7–15]. They showed that people with a lower SES visit general practitioner (GPs) more often and are more often hospitalized, whereas people with a higher SES use specialized care more often [7–9, 16]. There are several explanations for these differences in healthcare utilization. First, individuals with a lower SES generally have poorer health and higher co-morbidity, which contributes to more GP visits and more hospitalizations [17]. Second, individuals with a lower SES tend to have a lower income and more financial difficulties, which may restrict availability of certain health care services and deter them from seeking specialized care which tends to be more expensive [18]. Third, lower health literacy among lesser educated individuals may lead to difficulties in finding the appropriate health care and is shown to be related with suboptimal healthcare use [17, 18].

Injury is recognized as a leading cause of death and disability [19]. While several studies investigated the socio-economic differences in health care utilization in general and multimorbidity populations, less is known about these differences in trauma patients. Because of acuteness of the trauma and it’s recovery, health care utilization in the rehabilitation phase of trauma and the HRQoL of injury patients may differ from the general population and people with chronic conditions. Understanding health care utilization and HRQoL of educational groups may provide important insights into the inequalities following injury.

The present study therefore explores whether there are differences in health care utilization and HRQoL after trauma among different educational groups. The aims of this study were to a) compare the characteristics of trauma patients by educational level and to b) compare health care utilization and HRQoL of trauma patients by educational level.

## Methods

### Study population

This retrospective registry based cohort study was conducted with data from the Consumer Safety Institute’s Dutch Injury Surveillance System (DISS). DISS is a continuous monitoring system in which data from patients with injuries and/or poisoning visiting the Emergency Department (ED) of one of 14 hospitals throughout the Netherlands is collected [20]. The DISS registry is a representative reflection of ED visits in the Netherlands according to population characteristics with mix of academic and non-academic hospitals in both rural and urban areas. Age, sex, cause and type of injury and health care consumption during hospital admission are registered in DISS. The study does not fall under the Medical Research Involving Human Subject Act (non-WMO) (W16_394 # 17.004) as decided by the Medical Ethics Committee of the Academic Medical Center of Amsterdam (AMC), which means that no further ethical approval was required. Patients were informed on the DISS registry and could withdraw their data from the DISS registry at any time [20].

To get more insight into long-term consequences of injury and post-hospital health care utilization, the Consumer Safety Institute performed a follow-up study in a stratified sample of patients who visited the ED between January 2017 and December 2017, who had a place of residence in the Netherlands, unintentional injuries and survived hospital discharge. The study sample of injury patients aged 15 years and older, who visited an emergency department in the Netherlands, was stratified by type of injury (34 injury groups) and admission so that hospitalized patients and severe, less common injuries were overrepresented in the sample. Follow-up surveys were sent by mail to eligible patients 6 months after their ED visit. To increase the response rates, patients who did not respond to the survey, received a reminder. For the follow-up study, all participants signed a written informed consent form, which was included in the six-month follow-up questionnaire. In the present study, we included patients ≥25 years (as highest attained level of education is more stable from that age) and excluded respondents with unknown educational level.

### Data collection

#### Sociodemographic variables

Information on age, sex and injury-related characteristics (cause of injury, type of injury, number of injuries, hospitalization, length of hospital stay) were retrieved from DISS. Cause of injury was categorized as: home- and leisure related injuries, traffic injuries, sport injuries or occupational injuries. Injury groups were based on the EUROCOST classification [21]. In the DISS, up to three injuries can be recorded by type and body region. In case of multiple injuries, the primary injury was determined by an algorithm with priority to spinal cord injury, skull/brain injury, hip fracture and other lower extremity fractures over injuries in other body regions and to fractures over other injuries.

The 6-month follow-up survey included questions on age, sex, pre-injury health, marital status and level of education. Whether respondents identified as transgender was not measured in this study. Chronic conditions included respiratory diseases, heart disease, previous stroke, diabetes mellitus, hernia, (rheumatoid) arthritis, cancer and other. Patients were categorised into ‘no chronic conditions’, ‘one chronic condition’ and ‘two or more chronic conditions’. Marital status was categorised as: living together with a partner in the same household or living without a partner. Level of education was categorised according to the 2011 International Standard Classification of Education (ISCED) into low (primary school, lower secondary school or lower vocational training [0–2]), middle (intermediate and higher secondary school, or intermediate vocational training [3, 4], and high (higher vocational training or university education [5–8]) [22].

#### Health care utilization

The follow-up survey included questions on health care use. Patients reported on post-discharge intramural (e.g. stay at hospital or other institution) and extramural (e.g. homecare) medical care that was received because of their injury in the past 6 months. Patients were asked to report the number of specialist consultations, visits to a rehabilitation centre, to a general practitioner, to a physiotherapist, to a psychologist, and the number of weeks of receiving home care (e.g., nursing care, help with daily activities). In the Netherlands, GP, outpatient specialist care, outpatient rehabilitation care, and nursing care at home are covered by the mandatory health care insurance. Outpatient care consists of health care to persons who do not require all-day treatment.

#### Health-related quality of life

The follow-up survey included the EQ-5-Dimension, 5-Level (EQ-5D-5L) to assess HRQoL. This instrument consists of five items on different dimensions of health: mobility, self-care, usual activities, pain/discomfort, and anxiety/depression. There are five ordered response options: ‘no problems’, ‘slight problems’, ‘moderate problems’, ‘severe problems’ or ‘extreme problems’ [23]. The scores of the dimensions (1 to 5 in each dimension), can be converted into a health utility score. The Dutch value set was used to calculate the utility score ranging from 0 (death) to 1 (full health) [24]. The EQ-5D-5L also includes a visual analogue scale (EQ VAS), measuring the patient’s self-rated health, consisting of a scale ranging from 0 (worst imaginable health state) to 100 (best imaginable health state).

### Statistical analysis

Descriptive statistics were used to analyse patient and injury characteristics for the total sample as well as by educational level. Differences by educational level regarding patient characteristics were tested with Pearson Chi Square test for categorical variables, and with the one-way ANOVA test for continuous variables. A non-response analysis, regarding socio-demographic and injury characteristics, was conducted to determine whether responders differed from non-responders regarding age, sex, cause of injury, type of injury and length of hospital stay. Chi-square tests were used for categorical variables and independent sample t-tests for continuous variables. Descriptive health care utilization was reported in three categories: no, low or high health care utilization. Patients with at least one contact/visit were categorized into low and high health care utilization groups by median split. Differences by educational level regarding health care use per service were tested with the Pearson Chi Square test.

For the regression analysis, missing data was imputed for each educational level separately and combined afterwards [25]. Missing outcome data was not imputed. Missing data were imputed 10 times using multiple imputations by chained equations (MICE) to create 10 multiply imputed datasets [26]. The method used is based on fully conditional specification, where each incomplete variable is imputed by a separate model and combined using Rubin’s rules. Univariable and multivariable logistic regression analyses were used to estimate the association between education and the utilization (yes/no) of the following health services: outpatient specialist, outpatient rehabilitation, general practitioner, physiotherapist, psychologist, nursing care at home The following covariates were included in the logistic regression analysis: age, sex (male/female), comorbidity status (0, 1, ≥ comorbidities), marital status (living with or without a partner), cause of injury (home and leisure, traffic, occupational, sports), type of injury (head, thoracic, spinal cord, pelvic/hip, upper extremity, lower extremity), number of injuries (1, 2, ≥3) and length of hospital stay in days. Length of stay in hospital was used as a proxy for injury severity. Pooled results from the imputed datasets were used in the regression models and non-imputed data were used to perform the descriptive analysis. For comparison, complete case logistic regression analysis on the subset of complete cases were performed and outcomes were compared with results based on the imputed dataset. Descriptive statistics were used to analyse EQ-5D-5L utility scores and EQ VAS scores by educational level, for hospitalized and non-hospitalized patients. Differences by educational level regarding HRQoL were tested with the Pearson Chi Square test. A *p*-value lower than 0.05 was considered statistically significant. All analyses were performed in statistical package SPSS for Windows, version 25 (IBM SPSS Statistics, SPSS Inc., Chicago, IL) and R software (version 3.5).

## Results

### Patient population

**Fig. 1:**
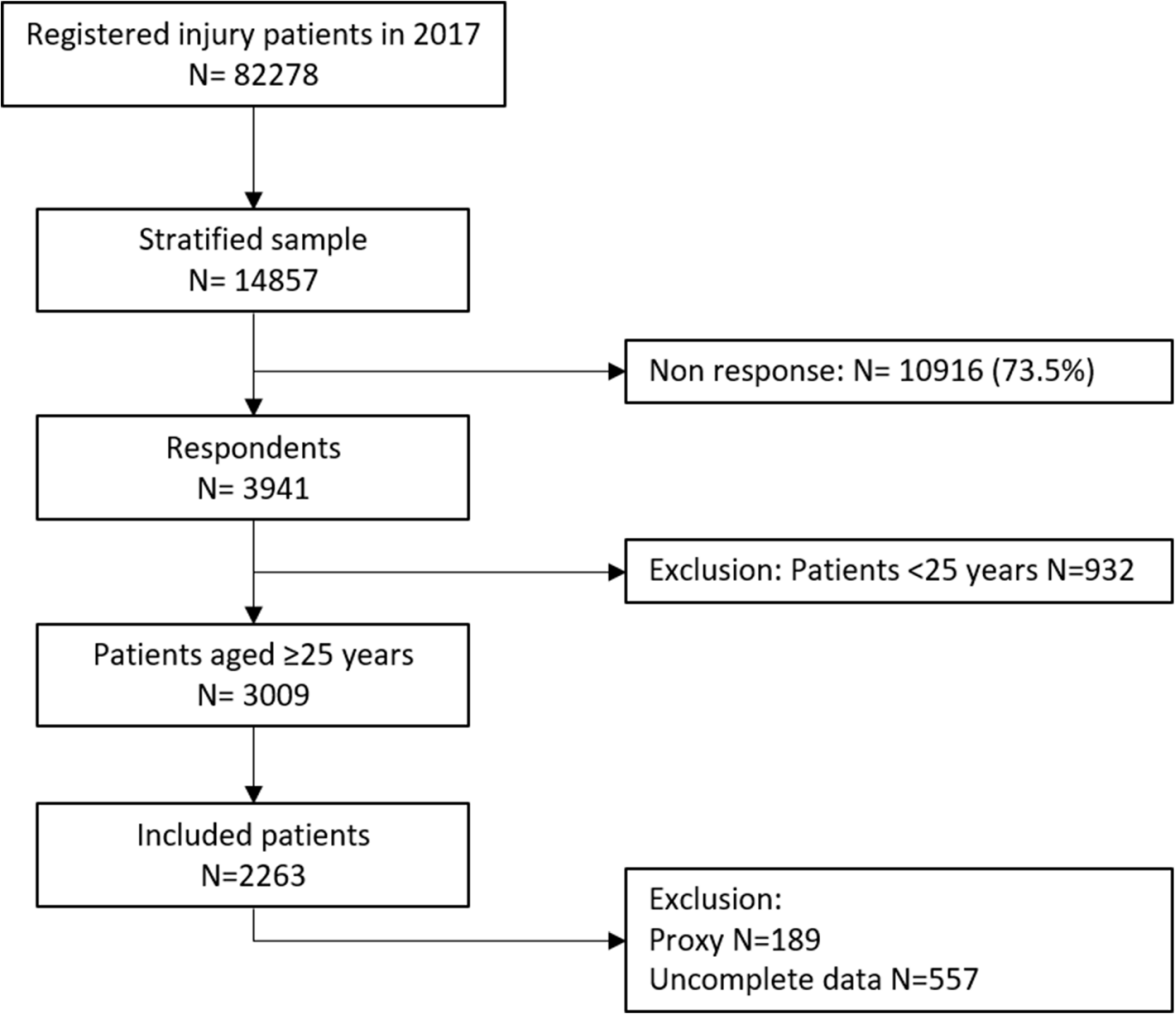
Flowchart of the study population

In total, 2606 patients aged ≥25 years were included in this study (Fig. 1). Non-response analysis showed that participants were significantly older, more often female and more often hospitalized than non-respondents (see Additional file 1). Educational level was low for 47.9% (*n* = 1249) of patients, middle for 24.4% (*n* = 635) of patients and high for 27.7% (*n* = 722) of patients. Slightly more than half of the sample were female (56.0%) and the mean age was 63.7 (SD 15.6) years (Table 1).

**Table 1:** Characteristics of study population

Characteristic	Total (***n*** = 2606)	Educational level			***p-***value
		Low (***n =*** 1249, 47.9%)	Middle (***n =*** 635, 24.4%)	High (***n =*** 722, 27.7%)	
Sex: Female, n (%)	1459 (56.0)	805 (64.5)	300 (47.2)	354 (49.0)	< 0.001
Age, mean (SD)	63.7 (15.5)	69.4 (12.9)	58.8 (15.6)	58.2 (16.2)	< 0.001
Comorbidity, n chronic conditions (%)^a^					
No	1372 (52.6)	576 (46.1)	357 (56.2)	438 (60.7)	< 0.001
One	725 (27.8)	356 (28.5)	181 (28.5)	188 (26.0)	0.454
More than one	437 (16.8)	272 (21.8)	84 (13.2)	81 (11.2)	< 0.001
Marital status: living with partner, n (%)^b^	1774 (68.1)	782 (62.6)	460 (72.4)	532 (73.7)	< 0.001
Cause of injury					
Home and leisure	1542 (59.2)	826 (66.1)	335 (52.8)	381 (52.8)	< 0.001
Traffic	650 (24.9)	293 (23.5)	165 (26.0)	192 (26.6)	0.236
Sport	257 (9.9)	48 (3.8)	79 (12.4)	130 (18.0)	< 0.001
Occupational	157 (6.0)	82 (6.6)	56 (8.8)	19 (2.6)	< 0.001
Type of injury, n (%)					
Mild TBI	201 (7.7)	86 (6.9)	51 (8.0)	64 (8.9)	0.268
Severe TBI	102 (3.9)	50 (4.0)	20 (3.1)	32 (4.4)	0.466
Other head or facial	211 (8.1)	92 (7.4)	54 (8.5)	65 (9.0)	0.779
Spinal cord injury	167 (6.4)	77 (6.2)	46 (7.2)	44 (6.1)	0.612
Rib fracture	98 (3.8)	46 (3.7)	30 (4.7)	22 (3.0)	0.264
Other thoracic injury	101 (3.9)	51 (4.1)	26 (4.1)	24 (3.3)	0.665
Fracture upper extremity	482 (18.5)	234 (18.7)	110 (17.3)	138 (19.1)	0.667
Other injury upper extremity	267 (10.2)	103 (8.2)	74 (11.7)	90 (12.5)	0.005
Pelvic injury	66 (2.5)	41 (3.3)	11 (1.7)	14 (1.9)	0.063
Hip fracture	265 (10.2)	158 (12.7)	58 (9.1)	49 (6.8)	< 0.001
Fracture of lower extremity	368 (14.1)	192 (15.4)	85 (13.4)	91 (12.6)	0.195
Other injury of lower extremity	244 (9.4)	106 (8.5)	61 (9.6)	77 (10.7)	0.270
Other	34 (1.3)	13 (1.0)	9 (1.4)	12 (1.7)	0.483
Hospitalized, n (%)^c^	1260 (49.7)	668 (55.5)	288 (46.8)	304 (43.2)	< 0.001
Hospital length of stay, mean (SD)^d^	5.6 (5.2)	6.1 (5.2)	4.9 (4.2)	5.0 (5.8)	< 0.001

*Abbreviations*: *SD* standard deviation, *TBI* traumatic brain injury

^a^73 missing values (2.8%)

^b^16 missing values (0.6%)

^c^73 missing values (2.8%)

^d^Patients with no contact not included

### Characteristics by educational level

Missing values existed for the confounding variables marital status (*n* = 16, 0.6%), comorbidity status (*n* = 73, 2.8%) and length of hospital stay (*n =* 73, 2.8%). Patients with a low educational level were more often female, were on average 11 years older, and lived more often without a partner compared to patients with a middle and high educational level (*p* < 0.001) (Table 1). Patients with a low educational level more often reported chronic conditions compared to patients with a high educational level (*p <* 0.001), Home and leisure related (*p <* 0.001) and occupational (*p <* 0.001) injuries were more common in patients with a low educational level, while sports related injuries were more common in patients with a higher educational level (*p <* 0.001). Hip fractures were significantly more common in patients with a low educational level (*p* < 0.001) while upper extremity injuries were more common in patients with a high educational level (*p* = 0.005). For all other type of injuries, no significant differences between educational groups were found. Patients with a low level of education (55.5%) were more likely to be admitted after trauma compared to those with a middle (46.8%) or high (43.2%) level of education (*p <* 0.001). And, if admitted, patients with a lower level of education had a significantly longer hospital stay (*p <* 0.001).

### Health care utilization by educational level

Table 2 reports the unadjusted six-month health care utilization for the total sample and for each educational group. A higher proportion of patients with a high education had at least one outpatient specialist visit, a GP visit, or a physiotherapist visit, while a higher proportion of patients with a low level of education had at least one outpatient rehabilitation visit, a psychologist visit, or received nursing care at home. Though, differences between educational levels were not significant, except for general practitioner visits and nursing care at home (*p* < 0.001). If a specific service was used, patients with a low educational level had on average more visits for all services studied, except for physical therapy.

**Table 2:** Six-month post-discharge health care utilization related to trauma for different health care services, by educational level

Characteristic	Total (***N =*** 2606)	Educational level			***p-***value
		Low (***n =*** 1249, 47.9%)	Middle (***n =*** 635, 24.4%)	High (***n =*** 722, 27.7%)	
Outpatient specialist visit, n (%) ^a^					0.768
None	907 (37.0)	435 (38.3)	224 (36.8)	248 (35.2)	
1–2 visits	847 (34.6)	385 (33.9)	209 (34.4)	253 (35.9)	
≥ 3 visits	695 (28.4)	317 (27.9)	175 (28.8)	203 (28.8)	
Mean (SD) number of visits^1^	3.2 (3.3)	3.4 (3.8)	3.2 (3.3)	3.0 (2.6)	
Outpatient rehabilitation n (%) ^b^					0.281
None	2215 (90.7)	1012 (89.3)	559 (91.6)	644 (92.1)	
1–5 visits	114 (4.7)	60 (5.3)	25 (4.1)	29 (4.1)	
≥ 6 visits	113 (4.6)	61 (5.4)	26 (4.3)	26 (3.7)	
Mean (SD) number of visits^1^	12.9 (20.0)	13.3 (20.4)	12.5 (14.4)	12.6 (23.6)	
General practitioner, n (%) ^c^					0.009
None	1635 (65.6)	795 (67.9)	392 (62.8)	448 (64.3)	
1–2 visits	621 (24.9)	259 (22.1)	165 (26.4)	197 (28.3)	
≥ 3 visits	236 (9.5)	117 (10.0)	67 (10.7)	52 (7.5)	
Mean (SD) number of visits^1^	2.4 (3.1)	2.6 (3.3)	2.3 (2.5)	2.3 (3.4)	
Physiotherapist, n (%) ^d^					0.986
None	1093 (45.2)	513 (45.6)	276 (45.6)	304 (44.3)	
1–10 visits	687 (28.4)	318 (28.2)	171 (28.3)	198 (28.8)	
≥ 11 visits	638 (26.4)	295 (26.2)	158 (26.1)	185 (26.9)	
Mean (SD) number of visits^1^	14.3 (12.4)	14.0 (11.9)	13.9 (11.8)	15.1 (13.5)	
Psychologist, n (%) ^e^					0.518
None	2396 (93.4)	1127 (92.5)	591 (93.7)	687 (94.6)	
1–3 visits	96 (3.7)	51 (4.2)	22 (3.5)	23 (3.2)	
≥ 4 visits	74 (2.9)	40 (3.3)	18 (2.9)	16 (2.2)	
Mean (SD) number of visits^1^	7.2 (11.5)	7.5 (11.6)	7.1 (10.4)	6.5 (12.4)	
Nursing care at home, n (%) ^f^					< 0.001
None	1948 (80.1)	814 (73.1)	520 (84.7)	614 (87.3)	
1–8 weeks	255 (10.5)	149 (13.4)	54 (8.8)	52 (7.4)	
≥ 9 weeks	228 (9.4)	151 (13.6)	40 (6.5)	37 (5.3)	
Mean (SD) number of weeks^1^	10.9 (8.2)	11.3 (8.2)	10.2 (7.9)	10.6 (8.7)	

*Abbreviations*: *GP* general practitioner, *SD* standard deviation, *IQR* inter quartile range

Patients with at least one contact/visit were categorized into low and high health care utilization groups by median split

^1^Patients with no contact not included

^a^73 missing values (2.8%)

^a^157 missing values (6.0%)

^b^164 missing values (6.3%)

^c^114 missing values (4.4%)

^d^188 missing values (7.2%)

^e^40 missing values (1.5%)

^f^175 missing values (6.7%)

Table 3 reports the unadjusted health care utilization by age group for each educational group. In general, differences between educational groups were similar over age groups. In the age group 25–44 years, the proportion of patients hospitalized (*p* < 0.001) and using outpatient rehabilitation (*p <* 0.001) was significantly higher in those with a low educational level. The higher proportion of home care in those with a low educational level was only significant in patients aged ≥65 years (*p <* 0.001). For patients aged 45–64, a significantly higher proportion of patients with a low education level visited a psychologist (*p* = 0.035). For all other services, no significant differences between educational levels were found.

**Table 3:** Hospitalization and 6-month post-discharge health care utilization related to trauma for different health care services, by age and educational level

Characteristic	Total	Educational level			***p-***value
		Low (***n*** = 1249, 47.9%)	Middle (***n*** = 635, 24.4%)	High (***n*** = 722, 27.7%)	
Hospitalization, n (%) ^a^					
25–44 years	106 (35.7)	23 (50.0)	43 (41.7)	40 (27.0)	0.005
45–64 years	397 (42.6)	152 (45.1)	125 (41.4)	120 (40.8)	0.490
≥ 65 years	757 (58.1)	493 (59.3)	120 (57.1)	144 (55.0)	0.438
Outpatient specialist visits, n (%) ^b^					
25–44 years	179 (60.9)	27 (60.0)	58 (56.9)	94 (63.9)	0.526
45–64 years	620 (67.2)	221 (67.4)	200 (67.3)	199 (67.0)	0.994
≥ 65 years	743 (60.3)	454 (59.4)	126 (60.3)	163 (62.7)	0.649
Outpatient rehabilitation, n (%) ^c^					
25–44 years	32 (10.7)	11 (25.0)	13 (12.6)	8 (5.3)	0.001
45–64 years	68 (7.3)	32 (9.4)	19 (6.3)	17 (5.8)	0.150
≥ 65 years	127 (10.5)	78 (10.4)	19 (9.3)	30 (11.9)	0.659
General practitioner visits, n (%) ^d^					
25–44 years	103 (34.0)	18 (38.3)	38 (36.2)	47 (31.1)	0.558
45–64 years	317 (34.0)	102 (30.1)	107 (35.3)	108 (37.4)	0.135
≥ 65 years	437 (34.7)	256 (32.6)	87 (40.3)	94 (36.6)	0.087
Physiotherapist visits, n (%) ^e^					
25–44 years	152 (51.5)	25 (55.6)	55 (53.9)	72 (48.6)	0.601
45–64 years	528 (57.4)	181 (54.2)	167 (56.6)	180 (61.9)	0.146
≥ 65 years	645 (53.6)	407 (54.5)	107 (51.4)	131 (52.8)	0.710
Psychologist visits, n (%) ^f^					
25–44 years	31 (10.3)	9 (19.1)	11 (10.5)	11 (7.2)	0.066
45–64 years	57 (6.0)	30 (8.6)	13 (4.2)	14 (4.7)	0.035
≥ 65 years	82 (6.3)	52 (6.3)	16 (7.3)	14 (5.2)	0.616
Nursing care at home, n (%) ^g^					
25–44 years	15 (5.0)	4 (8.5)	7 (6.7)	4 (2.7)	0.172
45–64 years	118 (12.5)	53 (15.7)	35 (11.4)	30 (10.1)	0.079
≥ 65 years	350 (29.4)	243 (33.3)	52 (25.9)	55 (21.3)	0.001

*Abbreviations*: *GP* general practitioner, *SD* standard deviation, *IQR* inter quartile range

^a^73 missing values (2.8%)

^b^157 missing values (6.0%)

^c^164 missing values (6.3%)

^d^114 missing values (4.4%)

^e^188 missing values (7.2%)

^f^40 missing values (1.5%)

^g^175 missing values (6.7%)

### Health care utilization by level of education adjusted for covariates

As outlined in Table 1, the characteristics between the three educational groups differed substantially. Therefore, associations between health care utilization and educational level were adjusted for important patient and injury characteristics (Table 4, Additional file 2)*.* Patients with higher education were more likely to visit a general practitioner (OR = 1.39, CI = 1.11–1.72) but less likely to be admitted to a hospital (OR = 0.80, CI = 0.63–1.00) and have nursing care at home (OR = 0.67, CI = 0.49–0.90) compared to patients in the low educational group. No significant differences between educational groups were found for other health care services studied. However, patients in the low educational group seemed to use slightly more outpatient rehabilitation care and psychological care and slightly less physical therapy compared to patients in the high educational group. Similar results were obtained when the analysis was restricted to complete cases only (see Additional file 3). Higher age was significantly associated with a higher likelihood of hospitalization and nursing care at home while associated with a lower likelihood of visiting a psychologist (see Additional file 2). While males were more often hospitalized and more often received rehabilitation care compared to females, they were less likely to visit a specialist, general practitioner, physical therapist, and less often received home care than females. Patients who reported one or more chronic diseases were more likely to visit the general practitioner and to receive homecare than patients without a chronic disease. Patients who lived with a partner were significantly less likely to visit a psychologist or to receive home care than patients living alone. Patients with pelvic/hip injury were most likely to be hospitalized compared to patients with other type of injuries. Additionally, patients with pelvic/hip and extremity injuries were most likely to visit a specialist or physical therapist but were less likely to visit the general practitioner. Length of hospital stay, as a proxy for injury severity, was associated with use of all health care services.

**Table 4:** Association between health care utilization (no/yes) and educational level, binary logistic regression analysis

	Hospitalization	6-month health care utilization after discharge^**a**^					
		Outpatient Specialist	Outpatient rehabilitation	General practitioner	Physiotherapist	Psychologist	Nursing care at home
	OR (95% CI)	OR (95% CI)	OR (95% CI)	OR (95% CI)	OR (95% CI)	OR (95% CI)	OR (95% CI)
**Unadjusted**							
Educational level (low)							
Middle	0.72* **(0.59;0.88)**	1.06 (0.87;1.30)	0.76 (0.54;1.08)	1.25* **(1.02;1.53)**	1.00 (0.82;1.22)	0.84 (0.57;1.23)	0.49* **(0.38;0.63)**
High	0.62* **(0.52;0.75)**	1.14 (0.94;1.39)	0.71* **(0.51;1.00)**	1.18 (0.96;1.43)	1.05 (0.87;1.28)	0.71 (0.48;1.05)	0.39* **(0.30;0.51)**
**Adjusted**^**b**^							
Educational level (low)							
Middle	0.87 (0.69;1.10)	1.07 (0.85;1.34)	0.92 (0.63;1.34)	1.41^*^ **(1.13;1.75)**	1.13 (0.90;1.42)	0.83 (0.55;1.26)	0.83 (0.61;1.12)
High	0.80* **(0.63;1.00)**	1.11 (0.89;1.39)	0.90 (0.62;1.31)	1.38^*^ **(1.11;1.72)**	1.19 (0.95;1.49)	0.76 (0.50;1.17)	0.66^*^ **(0.49;0.90)**

*Abbreviations*: *GP* general practitioner, *CI* confidence interval, *OR* odds ratio

^a^≥1 visit/contact within 6 months after injury

^b^Adjusted for age, sex, marital status, comorbidity status and cause of trauma, type of injury and number of injuries. 6-month health care utilization was additionally adjusted for length of hospital stay as proxy for injury severity.

^*^P<0.05

### Health-related quality of life by educational level

A mean EQ-5D-5L utility score of 0.76 (SD: 0.24) and an EQ VAS of 72.4 (SD: 18.7) was found for the total sample (Table 5, EQ-VAS scores see Additional file 4). The EQ-5D-5L utility score and EQ VAS score were highest (‘best’) for patients with a high educational level, respectively 0.82 (SD: 0.20) and 74.8 (SD: 17.5) and lowest (‘worst’) for patients in the low educational level, respectively 0.73 (SD: 0.26) and 70.3 (SD: 19.5) (*p* < 0.001).

**Table 5:** EQ-5D-5L utility scores by educational level

	Total	Educational level			***p***-value
		Low	Middle	High	
**EQ-5D-5L utility scores, mean (SD)**					
Total	0.76 (0.24)	0.73 (0.26)	0.78 (0.24)	0.81 (0.20)	<0.001
Age					
25-44	0.84 (0.19)	0.73 (0.27)	0.84 (0.19)	0.88 (0.13)	0.001
45-64	0.79 (0.23)	0.77 (0.23)	0.79 (0.23)	0.81 (0.21)	0.134
≥65 years	0.73 (0.26)	0.71 (0.27)	0.74 (0.26)	0.78 (0.22)	<0.001
Sex					
Male	0.80 (0.21)	0.77 (0.23)	0.81 (0.22)	0.83 (0.19)	<0.001
Female	0.73 (0.81)	0.70 (0.27)	0.74 (0.25)	0.79 (0.21)	<0.001
Type of injury					
Head	0.82 (0.22)	0.78 (0.24)	0.84 (0.23)	0.85 (0.19)	0.008
Spinal cord	0.71 (0.25)	0.66 (0.29)	0.73 (0.25)	0.79 (0.13)	0.039
Thoracic	0.83 (0.21)	0.77 (0.24)	0.88 (0.18)	0.88 (0.15)	0.011
Pelvic/hip	0.67 (0.25)	0.66 (0.25)	0.67 (0.27)	0.69 (0.25)	0.401
Upper extremity	0.80 (0.21)	0.78 (0.22)	0.79 (0.23)	0.84 (0.19)	<0.001
Lower extremity	0.72 (0.26)	0.67 (0.29)	0.75 (0.24)	0.79 (0.21)	<0.001
Number of injuries					
1	0.76 (0.24)	0.72 (0.26)	0.78 (0.24)	0.81 (0.20)	<0.001
2	0.77 (0.24)	0.73 (0.25)	0.79 (0.24)	0.82 (0.21)	<0.001
≥3	0.77 (0.23)	0.73 (0.26)	0.77 (0.24)	0.82 (0.17)	0.066
Hospitalization					
Not hospitalized	0.82 (0.22)	0.78 (0.24)	0.82 (0.22)	0.85 (0.17)	<0.001
Hospitalized	0.72 (0.26)	0.68 (0.27)	0.74 (0.25)	0.76 (0.22)	<0.001

*Abbreviation*: *SD* standard deviation

**Fig. 2:**
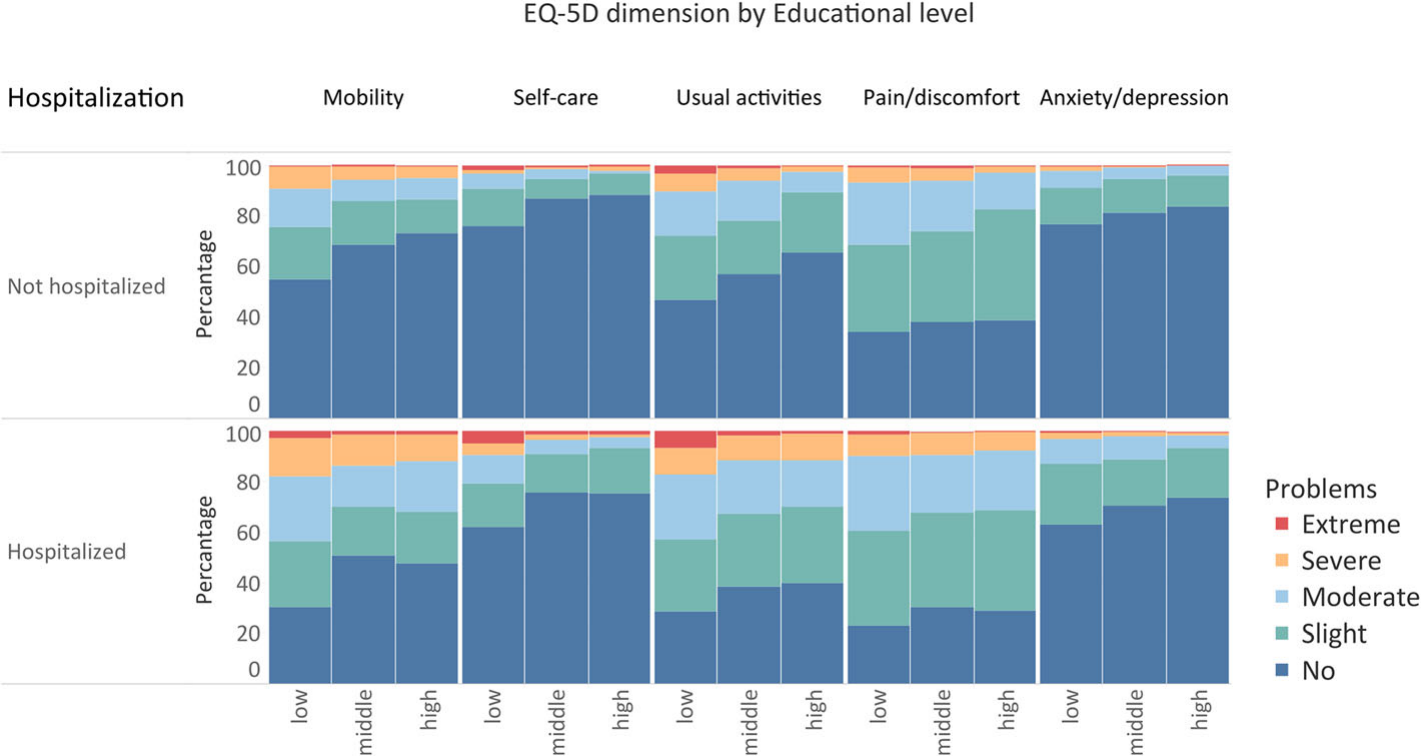
Frequency of EQ-5D-5L dimensions by educational level for hospitalized and non-hospitalized patients

In the total trauma population, problems were most frequently reported on the EQ-5D-5L dimension was ‘pain/discomfort’ (67.7%) while problems on the ‘self-care’ (23.9%) dimension were least frequently reported. Patients in the low educational group reported significantly more problems on all dimensions compared to patients in the middle and high educational group, although not statistically significant for non-hospitalized patients in the pain/discomfort dimension (Fig. 2). For non-hospitalized patients, largest differences between educational levels were reported for ‘usual activities’ (proportion of patients with problems by educational level: low 53.3%; middle 42.9%; high 34.6%) (*p* < 0.001). For hospitalized patients, the largest differences were found on the ‘mobility’ dimension (proportion of patients with problems by educational level: low 69.6%; middle 49.1%; high 52.3%) (*p <* 0.001).

## Discussion

The results of this study show that, in general, persons with a low educational level were more often hospitalized, more often received homecare and were less likely to visit the general practitioner compared to persons with high educational level. For most services, determinants other than educational level, including sex, age and injury related characteristics, were associated with the health care use. Additionally, this study shows that persons with a low educational level, rate their HRQoL lower than persons with a high educational level. For both hospitalized an non-hospitalized persons, those with low educational level reported lower HRQoL and more problems on all dimensions than those with middle and high educational level.

### Educational level and health care utilization

Consistent with existing literature, a larger proportion of patients with a low education were admitted for their trauma compared to patients with a middle or high level of education. And, if admitted, patients with a low education had on average a longer hospital stay [7, 9, 27, 28]. Some studies suggest that the higher use of acute care can be a result of a disrupted access to other healthcare services or inadequate health literacy in patients with a low education causing them for example to being less able to handle acute problems on their own [29–31]. However, more research is needed to identify factors that explain these differences between educational groups.

In the general population in the Netherlands and several other European countries, a trend can be observed where patients with a low educational level more often visit their GP [7–9, 32, 33]. Notably, our study showed opposite results compared to studies in general populations as higher educated patients visited the GP more often for injury-related problems in the 6 months after trauma compared to low educated patients. Possibly, higher educated patients are better informed about the reasons why they are supposed to follow-up with a GP after injury [34]. Another explanation could be that lower educated groups more often visit EDs as a substitute for primary care [35]. Additionally, lower educated patients in our population were generally older and more often already had nursing care at home. Therefore, they possibly have less need for general practitioner care.

Similar to other studies in general populations from both the Netherlands and other European countries [7–9, 32], trauma patients with a low educational level were more likely to receive nursing care at home. The higher age and the higher proportion of chronic diseases, may result in a slow or delayed recovery of their trauma, in patients with an low education compared to patients with a high education. A previous study showed for example that the rate of recovery following injury was slower for patients with multiple chronic diseases [36].

For outpatient specialist care, outpatient rehabilitation care, physical therapy, and psychological care, there were no significant differences between subgroups based on educational levels after correcting for patient and injury characteristics. However, our study showed that, in the rehabilitation phase of trauma, patients with a low education tend to use outpatient rehabilitation care more often and physical therapy less often compared to people with an middle and high education. A possible explanation for these findings is the pathway of care where hospitalization – more common in low educated patients – is often followed by outpatient rehabilitation care. This may be amplified by the Dutch health care system i.e., the GP, outpatient specialist care, rehabilitation care, and nursing care at home are covered by the mandatory health care insurance, while physical therapy requires additional insurance at extra cost or is paid out of pocket. This financial barrier could also be a reason why low educated patients were less likely to visit the physiotherapist after injury. Several studies from other European countries also foundan association between a higher educational level and higher physical therapy utilization in the general population [9, 37].

### Educational level and HRQoL

Low educational level was associated with lower HRQoL after injury which is in accordance with other studies on education and health outcome after trauma [38]. HRQoL was measured with the EQ-5D-5L and EQ VAS and mean EQ-5D-5L utility and EQ VAS scores were substantially lower in all educational groups than the scores for the general Dutch population [24]. Although data on pre-injury HRQoL in our population was not collected, this could indicate that patients are still recovering from their injury after 6 months. Other studies also showed that the majority of trauma patients had lower HRQoL scores 1 year post injury compared to their pre-injury health status or the general population [39, 40]. It is unclear whether the disparities in HRQoL were already present before trauma and in what way the trauma, recovery process influenced the HRQoL. The large differences by educational level on the ‘mobility’ dimension of the EQ-5D, could be explained by the general age difference between patients in the low educational group compared to those in the middle and high educational group. While multiple studies have established the relationship between higher educational attainment and HRQoL, it is important to further investigate the factors underlying this relationship.

### Strengths & limitations

It is important to gain a better understanding of health care use and health disparities in the trauma population, to identify strategies to ensure equal access to care, delivery of care and health outcome after trauma. Our study provided an opportunity to study educational differences in a trauma population with detailed data on both health care utilization and HRQoL. A multiple imputation approach was used to reduce bias from missing data. Sensitivity analysis was done on a complete case dataset and showed that outcomes and conclusions remained largely the same. However, there are several limitations that should be considered. First, in this study we only had data on education. Ideally, other dimensions as income and occupation could be included to provide additional information on the socio-economic status of the patient. These measures provide additional information for example on possible financial barriers for specific types of care (e.g. physical therapy). Additionally, patients with lower education were on average 10 years older than those with higher education, since most of today’s older population have no or low academic qualifications. Second, data on health care utilization and HRQoL was collected at the same time and pre-injury HRQoL was unknown. It would be valuable to investigate longitudinal changes in HRQoL to study cause-effect relations. Third, the respondents were not a representative sample of the patients registered in the DISS. Severe and less common injuries were intentionally overrepresented. Additionally, responders were significantly older, more often female and more often hospitalized. This may limit the generalizability of the results. Fourth, patients with a lower educational level were less likely to complete the questionnaire and the respondents with a lower educational level that did complete the questionnaire may not be representative of Dutch injury patients with a low educational level (e.g. with regards to health literacy). As a result, our findings may underestimate the differences in health care utilization between educational groups. Finally, the abbreviated injury scale (AIS) to assess the injury severity was not captured in the DISS and subsequently not included in our analysis. Therefore, hospital length of stay was used as a proxy for injury severity [41].

## Conclusion

Individuals with a low educational level are an important group for injury prevention, as our results show that the majority of injury patients have a low educational level with more hospitalization and poorer health outcome among these individuals. However, other factors including age and sex may also explain a part of the differences between educational groups. It is important that patients are aware of potential consequences of their trauma and when and why they should consult a specific health care service after ED or hospital discharge. Future research could examine whether health care needs of injury patients in all educational groups are met and/or examine if there are barriers for health care access and use for specific groups. This will shed light on factors that drive health care utilization of different education groups and the poorer health outcomes for patients with a low educational level.

## Supplementary Information

**Additional file 1.** Non-response analysis for patients aged ≥25 years. Description: Non-response analysis for patients aged ≥25 years

**Additional file 2.** Univariable and multivariable logistic regression analysis showing the determinants of health care utilization. Description: Univariable and multivariable logistic regression analysis

**Additional file 3.** Complete case association between health care utilization (no/yes) and educational level, binary logistic regression analysis. Description: Logistic regression analysis with complete cases

**Additional file 4.** EQ-VAS scores by educational level. Description: EQ-VAS scores by educational level and patient and injury characteristics

## Data Availability

The datasets generated and/or analysed during the current study are not publicly available due to information that could compromise the privacy of research participants. The datasets used and/or analysed during the current study are available from Martien Panneman (m.panneman@veiligheid.nl) on reasonable request.

## Abbreviations

HRQoL: Health related quality of life
EQ-VAS: EuroQol Visual Analogue Scale
EQ-5D-5L: 5-level EQ-5D version
SES: Socio-economic status
GP: General practitioner
DISS: Dutch Injury Surveillance System
ED: Emergency department
Non-WMO: Medical Research Involving Human Subject Act.
ISCED: International Standard Classification of Education
